# A high-throughput screening assay based on automated microscopy for monitoring antibiotic susceptibility of *Mycobacterium tuberculosis* phenotypes

**DOI:** 10.1186/s12866-021-02212-3

**Published:** 2021-06-05

**Authors:** Sadaf Kalsum, Blanka Andersson, Jyotirmoy Das, Thomas Schön, Maria Lerm

**Affiliations:** 1 Division of Inflammation and Infection, Lab 1, floor 12, Department of Biomedical and Clinical Sciences, Faculty of Medicine and Health Sciences, Linköping University, SE-58185 Linköping, Sweden; 2 Division of Clinical Microbiology, Department of Biomedical and Clinical Sciences, Faculty of Medicine and Health Sciences, Linköping University, SE-58185 Linköping, Sweden

**Keywords:** Cording, Planktonic, *Mycobacterium tuberculosis*, Whole-cell screening, Automated live-cell imaging

## Abstract

**Background:**

Efficient high-throughput drug screening assays are necessary to enable the discovery of new anti-mycobacterial drugs. The purpose of our work was to develop and validate an assay based on live-cell imaging which can monitor the growth of two distinct phenotypes of *Mycobacterium tuberculosis* and to test their susceptibility to commonly used TB drugs.

**Results:**

Both planktonic and cording phenotypes were successfully monitored as fluorescent objects using the live-cell imaging system IncuCyte S3, allowing collection of data describing distinct characteristics of aggregate size and growth. The quantification of changes in total area of aggregates was used to define IC_50_ and MIC values of selected TB drugs which revealed that the cording phenotype grew more rapidly and displayed a higher susceptibility to rifampicin. In checkerboard approach, testing pair-wise combinations of sub-inhibitory concentrations of drugs, rifampicin, linezolid and pretomanid demonstrated superior growth inhibition of cording phenotype.

**Conclusions:**

Our results emphasize the efficiency of using automated live-cell imaging and its potential in high-throughput whole-cell screening to evaluate existing and search for novel antimycobacterial drugs.

**Supplementary Information:**

The online version contains supplementary material available at 10.1186/s12866-021-02212-3.

## Background

Tuberculosis (TB) stands out as one of the most prevalent disease worldwide with a medication period significantly longer than that of other bacterial infections. The treatment of TB requires 6–9 months of chemotherapy with multiple drugs and is hampered by spread of antibiotic resistance, narrowing the therapeutic options. Conventional anti-TB drugs have been in use for many decades and the need to broaden their palette is urgent, pushed by an increase in incidence of multidrug-resistant and extensively drug-resistant TB cases. The search for new treatment regimens has resulted in the identification of several candidates, including new compounds and repurposed drugs [1].

Prolonged treatment and variable susceptibility to antibiotics can be attributed to the heterogeneity of populations of *Mycobacterium tuberculosis* (Mtb, the causative agent of TB). Diverse phenotypes exist both *in vitro* and *in vivo*, typically in infected lungs, where non-culturable, drug-tolerant bacteria can be found along with drug-susceptible [2]. The inherent ability of Mtb to form organized aggregates has been known for several decades [3] and has been often related to virulence. Cording mycobacteria grow aligned into tight bundles, where the orientation of the long axis of each bacterial cell within the cord is parallel to the long axis of the cord [4, 5]. As we have previously demonstrated, the cording phenotype represents more intrusive interaction with immune cells causing macrophages to release macrophage extracellular traps (METs) [6]. Recently, the cording phenotype has been shown to cause extensive immunopathological changes associated with active TB in C3HeB/FeJ mice [7]. Mtb cords were also identified inside human alveolar macrophages obtained from patients with active TB [8] and lymphatic endothelial cells isolated from patients with extrapulmonary TB [9]. Knowledge of the heterogeneity of mycobacterial populations increases in properly designed experimental setups [10] and is gaining increased attention in drug discovery models as currently reviewed by Parish [11].

Phenotypic drug screening based on the response of growing bacterial cultures allows the identification of a broader spectrum of inhibitors regardless on mechanism of their action. This so-called “whole-cell screening” has an advantage over target-based screening in that only molecules which can penetrate and actually kill/prevent growth of bacteria are selected as hits [12, 13]. This approach led to the discovery of bedaquilin, one of the few recently introduced anti-TB drugs [14]. More efficient methods based on reporter strains, such as H37Rv carrying bioluminescent or fluorescent genes, have facilitated the discovery of several leading compounds [15]. Due to the phenotypic heterogeneity of mycobacteria, where different metabolism and antibiotic susceptibility may coexist in the host [16], the need for combination of several anti-microbial drugs with different modes of action will continue to be a cornerstone in TB treatment. High-throughput assays allowing pairwise combinations can generate essential information for mathematical models to predict high-order interactions [17]. Image-based assays bring another dimension into drug screening and when automated systems are used in conjunction with live cultures, data reflecting the growth kinetics and morphological appearance of the models can be feasibly collected. The power of imaging assays for drug discovery based on investigating the intracellular fate of Mtb treated with anti-microbial compounds has been demonstrated in multiple studies [18–26]. However, axenic mycobacterial cultures can serve as a useful complement for deciphering the susceptibility of the extracellular phase of Mtb, such as observed within intact or rupturing granulomas or in cavitary TB [27, 28] and to our knowledge this model have not been analysed using imaging assays.

Given the evidence that cording represents an important intracellular phenotype and considering the potential importance of various phenotypes in compound discovery, we developed and validated an assay based on automated live-cell imaging to monitor growth of two distinct phenotypes of Mtb and used it in a checkerboard approach to analyse the effect of combinations of commonly used as well as recently developed TB drugs.

## Results

### Distribution of aggregate sizes reflects the bacterial phenotype and is altered during bacterial growth

**Fig. 1:**
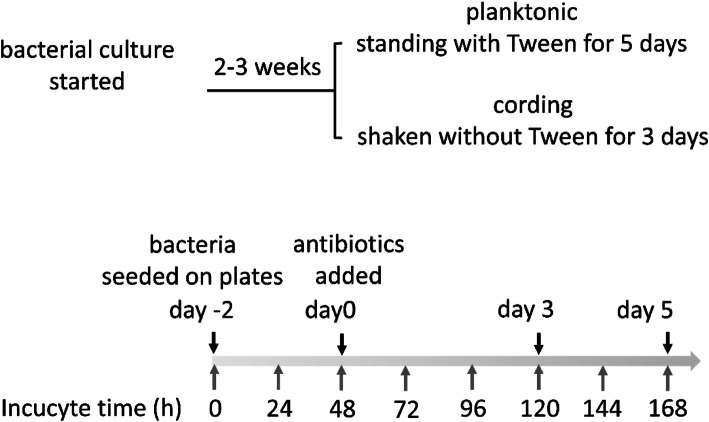
Scheme of the experimental layout

**Fig. 2:**
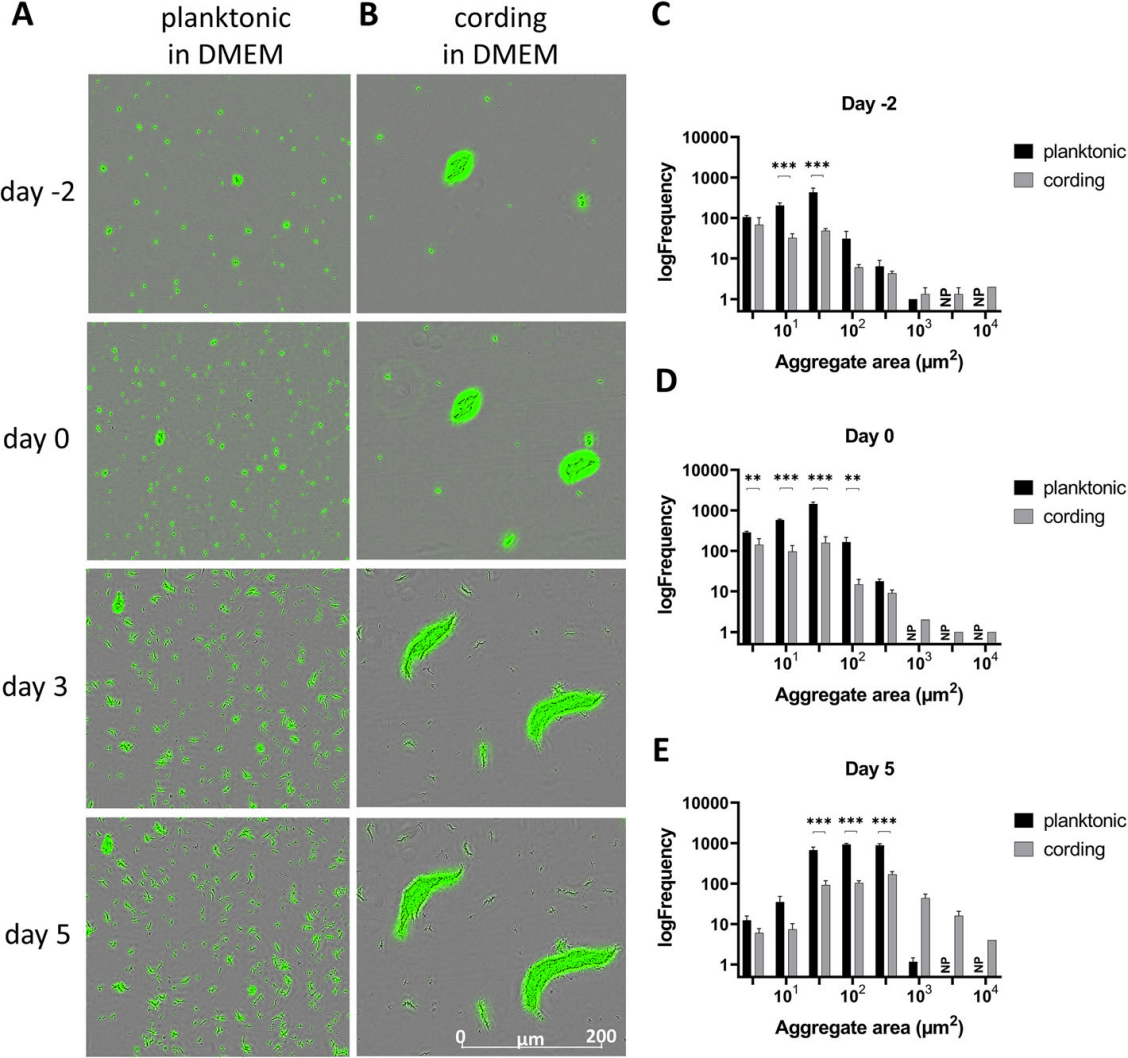
Morphological appearance and size of aggregates in planktonic and cording cultures. Phase contrast images (20x) of Mtb growing in DMEM as planktonic (**a)** and cording (**b**) bacteria are shown at different time points. Frequency plots represents the distribution of aggregate sizes at day − 2 (**c**), day 0 (**d**) and day 5 (**e**). Bars represent size intervals and are logarithmically distributed up to 10^4^ µm^2^. NP (non-present) marks intervals where no aggregates were identified. Data is presented as mean of log_10_ frequency ± SD (*n* = 3). Number of events in each experiment > 100. Significant differences between planktonic and cording phenotype are indicated with **(*p* ≤ 0.01), or ***(*p* ≤ 0.001) as determined by multiple t-tests with Holm-Sidak correction for multiple testing. Images were made by Incucyte® Base Software, version 2019B Rev3 (https://www.essenbioscience.com) and graphs by GraphPad Prism 9, version 9.0.0 (https://www.graphpad.com)

According to our previously published method [6] we obtained planktonic bacteria from standing cultures (Fig. 1) while bacteria more representative of the cording phenotype was obtained from Tween-80-free, shaken cultures (Fig. 1). Growth of both phenotypes expressing green fluorescent protein (GFP) were then followed in the live-cell imaging system IncuCyte S3, which enables automated collection of both phase contrast and fluorescent images in a high-throughput format. In line with our previous characterization of planktonic and cording phenotypes using scanning electron microscopy [6], bacteria generated in the standing culture were growing as small, dispersed aggregates (Fig. 2a), while the shaken culture resulted in more organized structures characteristic for mycobacterial cording (Fig. 2b). These phenotypic differences persisted throughout the experiment although the growth conditions were identical for both phenotypes (Tween-80-free cell culture medium) from the initiation of the experiment (Fig. 2a-b). In a parallel experiment, bacteria originating from standing cultures were seeded in wells with Middlebrook 7H9 broth with or without Tween-80. Again, the absence of Tween-80 promoted the cording phenotype (Additional file 1: Figure S1). We reasoned that the use of cell culture medium, in our case Dulbeccos´s Modified Eagle Medium (DMEM) with human serum instead of conventional broth, could be an advantage if our assay would be later optimized for drug screening using infected human cells. To further characterize both phenotypes, we measured the areas of fluorescent bacterial aggregates, categorized them into size intervals and presented in frequency plots (Fig. 2c-e). We defined day 0 as time when antibiotics were added to the bacterial suspension in later experiments. Distribution of aggregate sizes differed already in freshly harvested cultures on day − 2. In comparison to cording phenotype, planktonic contained significantly more small-sized aggregates, with no aggregates larger than 1000µm^2^ (Fig. 2c, Additional file 2: Table S1). A similar distribution was observed on day 0 (Fig. 2d, Additional file 2: Table S1). On day 5 both phenotypes showed an increased frequency of larger aggregates (Fig. 2e, Additional file 2: Table S1) apparently due to the growth of bacteria as enlarging aggregates rather than as separated cells.

A limitation of the IncuCyte software (2019B Rev3) is that it cannot be used to determine the size of a selected aggregate over time. To circumvent this problem, we wrote a MATLAB script to create simulations based on images captured by the instrument in a time course. The MATLAB image processing tool allowed us to follow the growth of individual aggregate (Additional file 3: Movie S1) and to estimate changes in area, reflecting aggregate growth (Additional file 4: Figure S2).


**Additional file 3: Movie S1.** Analysis of single aggregate in MATLAB. Growth of single aggregate was followed by MATLAB image processing tool. Movie shows the growth of an original object (background) used for real pixel acquisition generated from the IncuCyte® using the full frame pixel acquisition. Red mask, surrounding the aggregate in the upper-left corner of the image (inset 1), represents segmentation of the original object as generated using the MATLAB tool (segmented values are showed as X and Y). Figure in lower-left corner (inset 2) visualizes the increase in area (in pixels) overtime. Images were made by Incucyte® Base Software, version 2019B Rev3 (https://www.essenbioscience.com) and MATLAB (v R2017a, https://se.mathworks.com).

### Distribution of aggregate sizes reflects antibiotic exposure

**Fig. 3:**
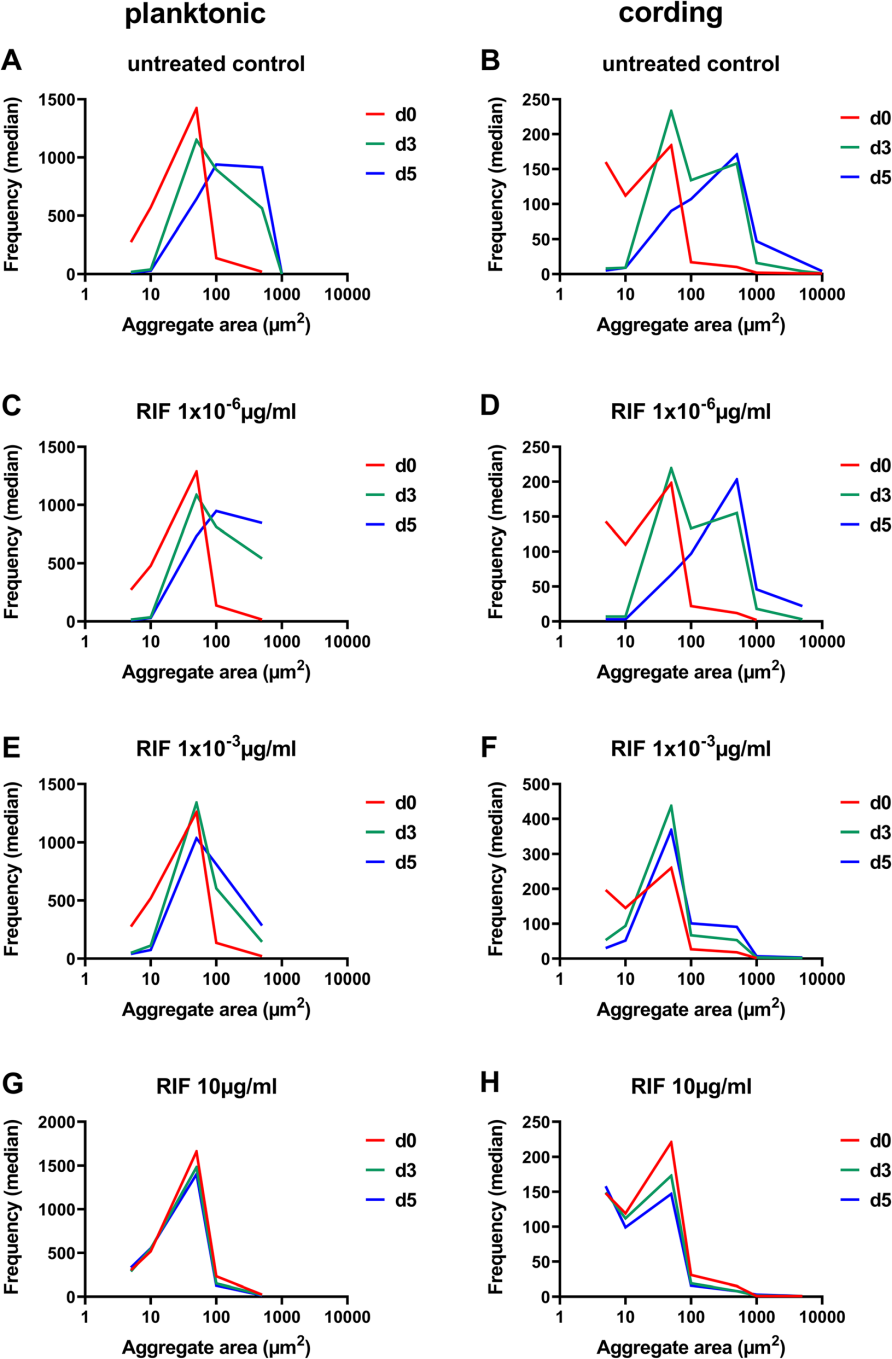
Frequency of aggregate sizes during bacterial growth and in response to rifampicin (RIF). Planktonic and cording phenotype were left untreated (**a**-**b**) or exposed to increasing concentration of rifampicin (**c**-**h**). Data is presented as median of frequencies of aggregates sizes in each interval (*n* = 3). Number of events in each experiment > 100. Graphs were made by GraphPad Prism 9, version 9.0.0 (https://www.graphpad.com)

**Fig. 4:**
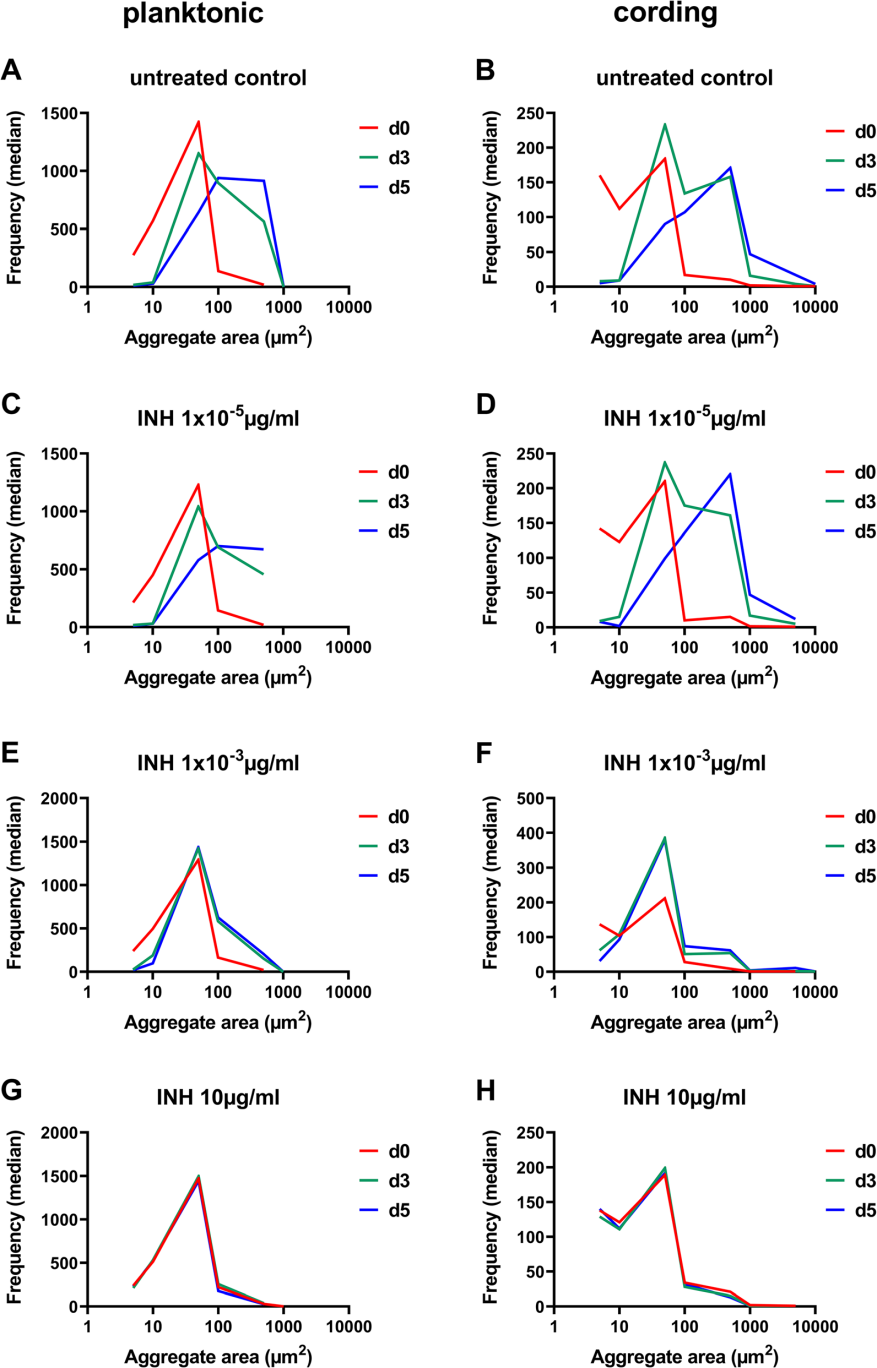
Frequency of aggregate sizes during bacterial growth and in response to isoniazid (INH). Planktonic and cording phenotype were left untreated (**a**-**b**) or exposed to increasing concentration of isoniazid (**c**-**h**). Data is presented as median of frequencies of aggregates sizes in each interval (*n* = 3). Number of events in each experiment > 100. Graphs were made by GraphPad Prism 9, version 9.0.0 (https://www.graphpad.com)

To investigate whether the distribution of aggregates sizes would be affected by antibiotic exposure, we treated cultures with different concentrations of rifampicin (RIF) and isoniazid (INH) and plotted median frequencies of aggregates in each size interval over time (Figs. 3 and 4). For both planktonic and cording phenotypes, untreated bacteria as well as those with the lowest concentration (1 × 10^− 6^ µg/ml) of RIF showed a shift over time towards higher number of aggregates with larger areas (Fig. 3a-d), indicating the growth of the aggregates. A moderate shift in frequencies was observed in wells treated with an intermediate concentration of RIF (1 × 10^− 3^ µg/ml, Fig. 3e-f). In contrast, no such shift could be observed when bacteria were treated with the highest concentration of RIF (10 µg/ml) indicating growth inhibition (Fig. 3g-h). Similar results were observed for bacteria treated with INH (Fig. 4a-h). Additionally, we compared average area of the aggregates between planktonic and cording phenotype and its change over time. Cording phenotype contained significantly larger aggregates then planktonic phenotype at day 5 when bacteria were left untreated or were treated with low concentration of RIF or INH (Additional file 5: Figure S3). At the higher concentrations, no differences in aggregate areas were observed at the later time points. This indicates that higher growth of cording phenotype was suppressed by the antibiotics.

### IC_50_ and MIC values show modest difference between planktonic and cording phenotype in susceptibility to antibiotics

**Fig. 5:**
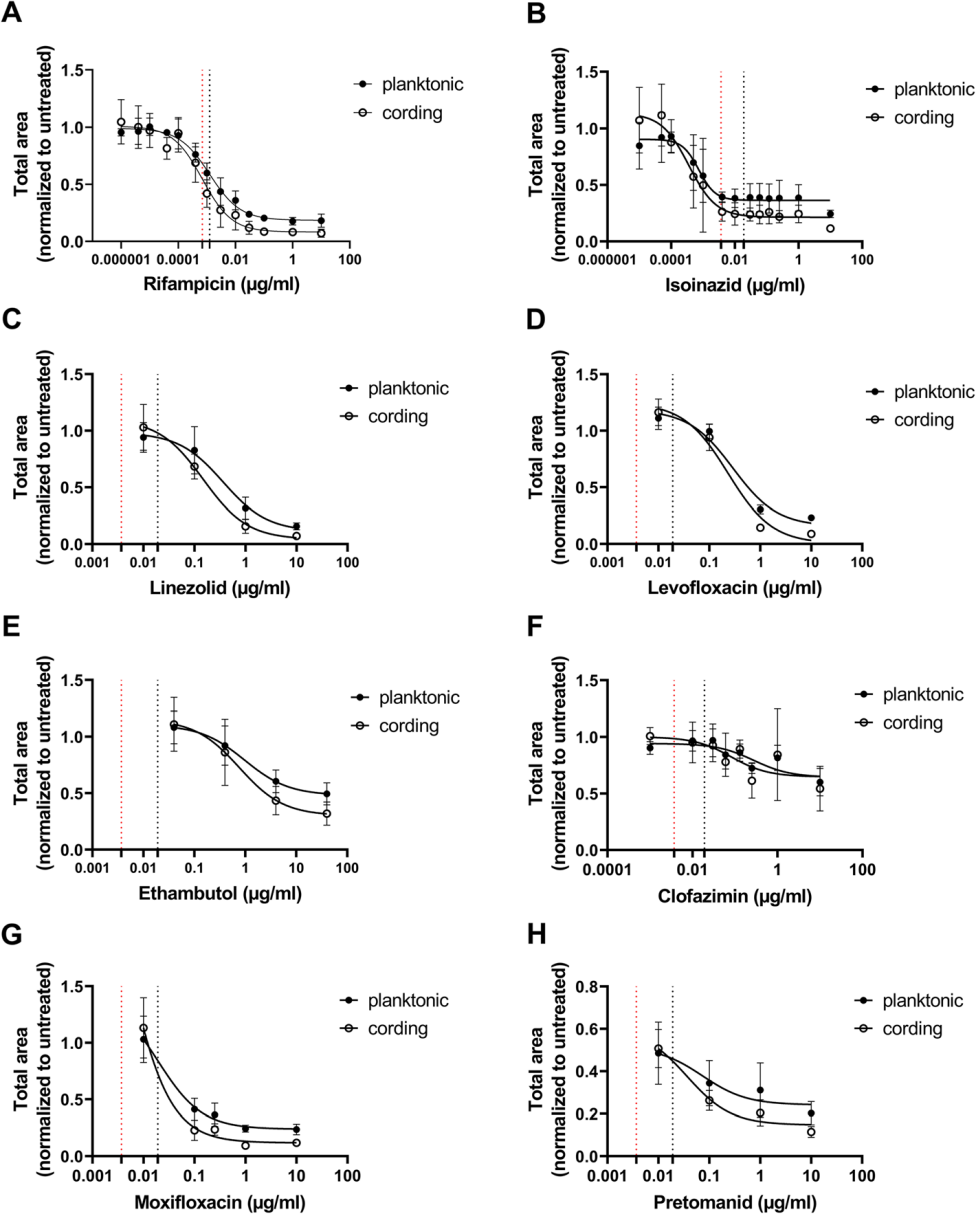
Dose response to antibiotics. Planktonic and cording bacteria were treated with antibiotics at several stepwise-diluted concentrations. IC_50_ values were determined by nonlinear regression with 4 parameters for RIF and INH (**a**-**b**) or 3 parameters for other antibiotics with less concentrations tested (**c**-**h**). Dotted lines cross the x-axes at the points representing IC_50_ value for planktonic (black line) and cording (red line) phenotype. Data is presented as mean total area after antibiotic exposure normalized to untreated control ± SD (*n* = 3). Graphs were made by GraphPad Prism 9, version 9.0.0 (https://www.graphpad.com)

**Fig. 6:**
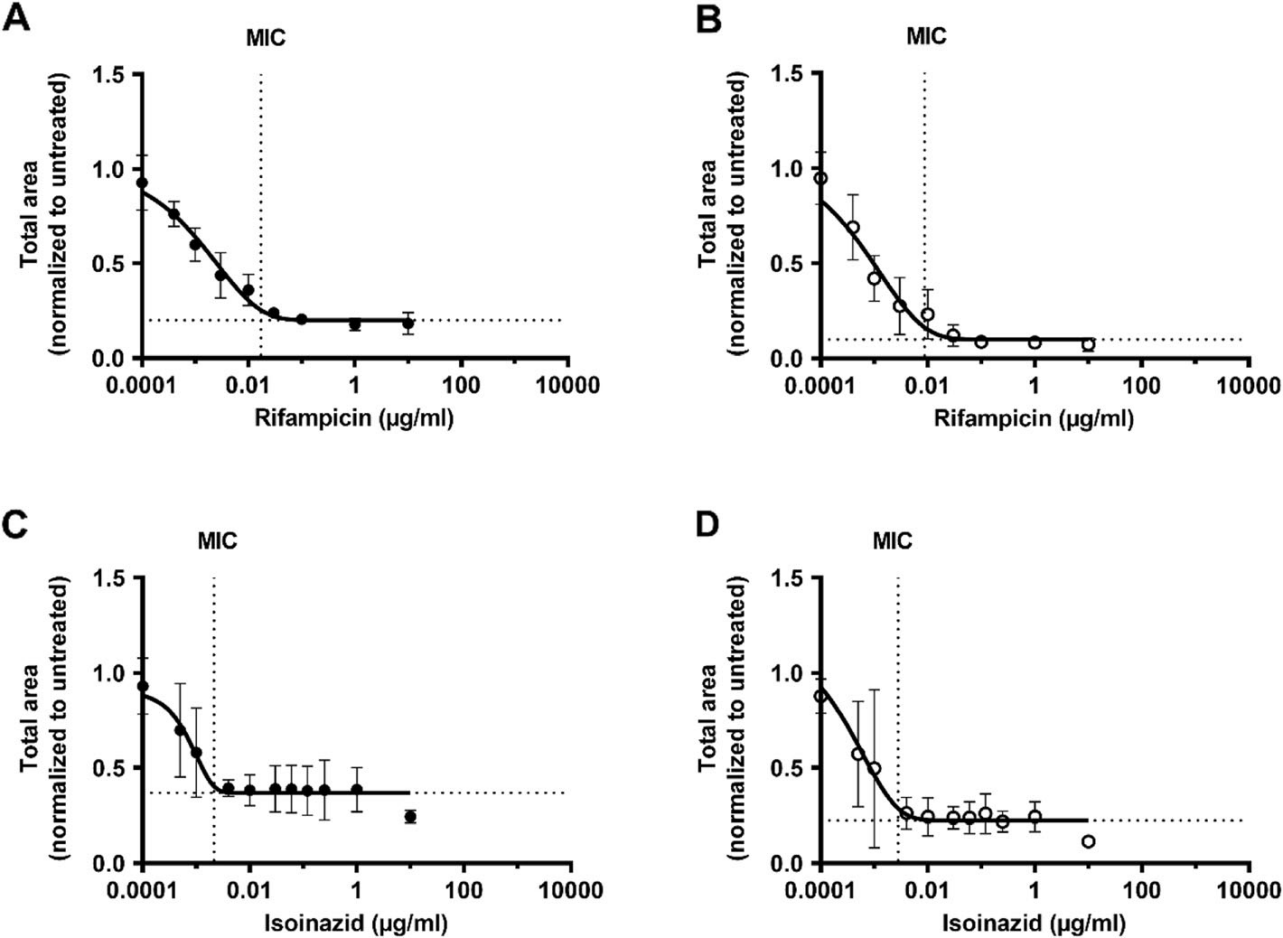
MIC values for rifampicin (RIF) and isoniazid (INH). Planktonic (**a**, **c**) and cording (**b**, **d**) bacteria were treated with 13 stepwise-diluted concentrations of RIF and INH. MIC values was determined at day 5 using modified Gompertz function. Vertical dotted lines cross the x-axes at the MIC value and horizontal dotted lines mark the bottom plateau of the curves. Data is presented as mean total area after antibiotic exposure normalized to untreated control ± SD (*n* = 3). Graphs were made by GraphPad Prism 9, version 9.0.0 (https://www.graphpad.com)

**Fig. 7:**
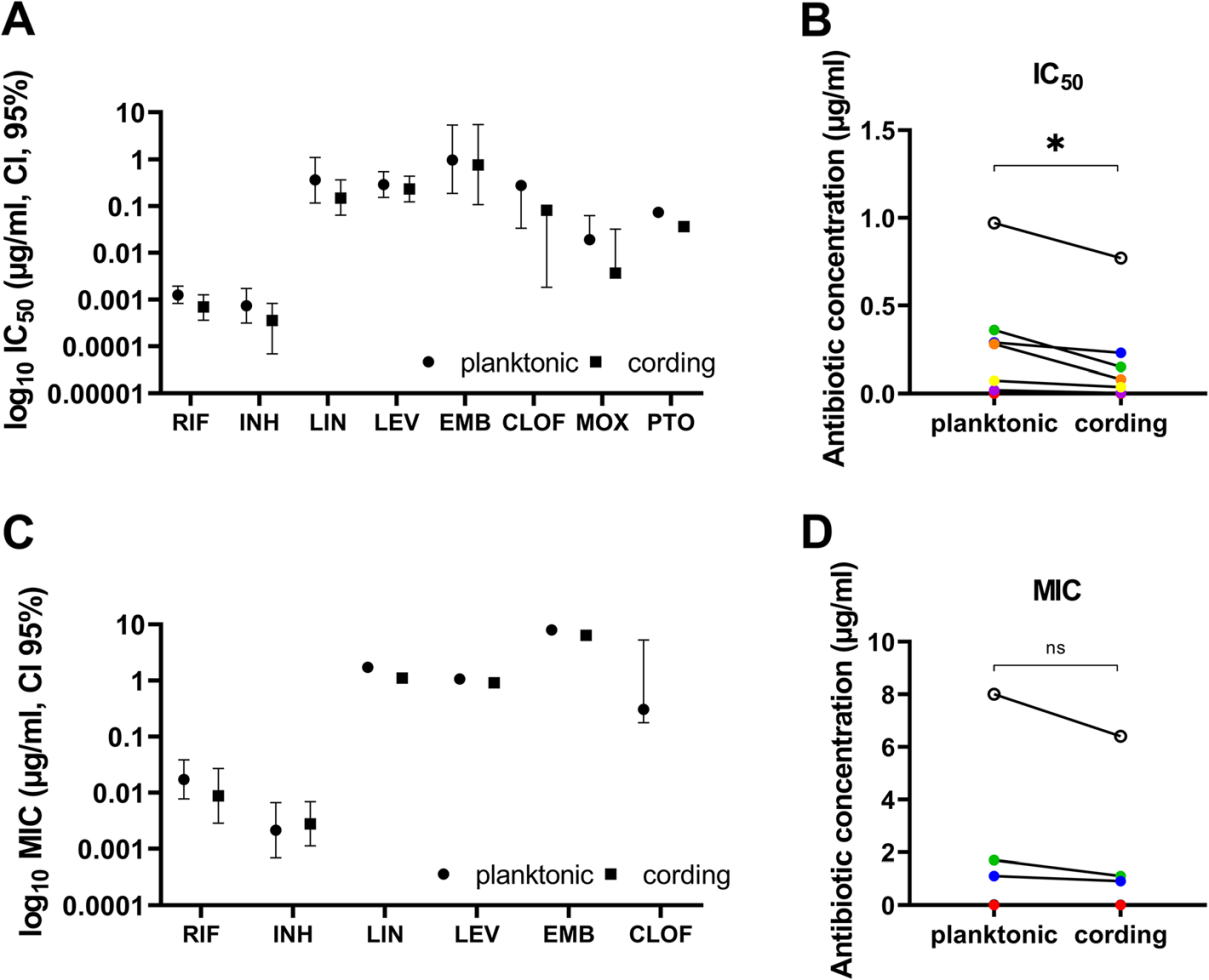
Comparison of differences in IC_50_ and MIC values for all antibiotics. IC_50_ and MIC values were compared between planktonic and cording phenotypes for each individual antibiotic (**a** and **c**) and all antibiotics combined (**b** and **d**). Data is presented as concentration (µg/ml) ± CI (95 %) determined by nonlinear regression for IC_50_ and Gompertz function for MIC (*n* = 3). Significant difference between planktonic and cording phenotype is indicated with *(*p* ≤ 0.05) and non-significant difference with ns as determined by paired, two-tailed t-test. Isoniazid (black), rifampicin (red), linezolid (green), levofloxacin (blue), ethambutol (white), clofazimine (orange), moxifloxacin (violet) and pretomanid (yellow). Graphs were made by GraphPad Prism 9, version 9.0.0 (https://www.graphpad.com)

To better evaluate whether there was a difference in antibiotic susceptibility between the two studied phenotypes, we determined the Inhibitory Concentration 50 (IC_50_) and Minimal Inhibitory Concentration (MIC) of RIF and INH as well as other first- and second-line TB antibiotics (Table 1) for the respective phenotype. The total area of fluorescent objects per image was measured. Intra-assay variability of measurements at day 5 in each of three experiments was 6,1 %, 16,1 % and 8,2 % for the planktonic and 10,6 %, 13,6 % and 17,8 % for the cording phenotypes. Inter-assay variability based on data from all three experiments was 10,1 % for the planktonic phenotype and 19,9 % for the cording phenotype (Additional file 6: Table S2). As the initial inoculum and growth rate differed significantly between the two phenotypes (Additional file 7: Figure S4), we normalized the measurements after antibiotic treatment to the untreated controls for each phenotype separately (Additional files 8 and 9: Figure S5-S6). We extracted day 5 data from cultures treated with 13 stepwise diluted concentrations of RIF and INH (Figs. 5 and 6). Data revealed that the cording phenotype was slightly more susceptible to RIF than the planktonic, with both IC_50_ and MIC values (Table 1; Figs. 5a and 6a-b) being twice as high. Cording bacteria also showed a higher susceptibility to INH over planktonic when comparing IC_50_ values (Table 1; Fig. 5b), but there was no difference in MIC values (Table 1; Fig. 6c-d). Significance was not reached in statistical comparison of the susceptibility of the two phenotypes when the studied antibiotics were analyzed separately (Table 1; Fig. 7a, c). However, when combining the results from all antibiotics tested, IC_50_ values were significantly lower for cording compared to planktonic phenotype (Fig. 7b), whereas no significant difference was found in MIC values (Fig. 7d). This was also true after transposition of MIC values to nearest higher MIC according to ISO standard (Table 1).

**Fig. 8:**
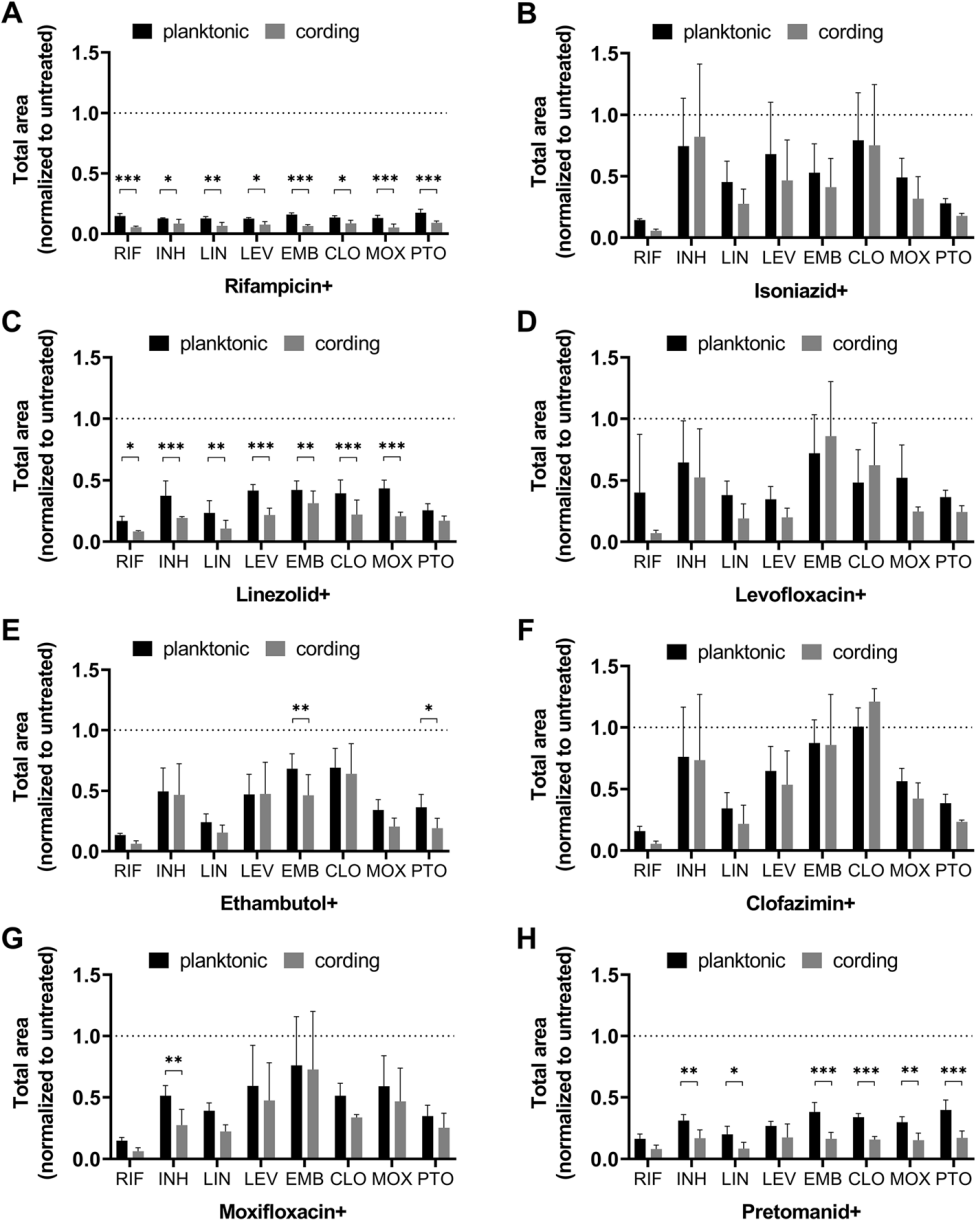
Inhibition of Mtb growth by combination of antibiotics. Planktonic and cording phenotypes were treated by a combination of two antibiotics at sub-inhibitory concentrations (details in Table 2). Data is presented as mean total area normalized to untreated control ± SD (*n *= 3). Dotted line marks the level of uninhibited growth in controls to which data after exposure to antibiotics were normalized. Significant differences between planktonic and cording phenotype are indicated with *(*p* ≤ 0.05), **(*p* ≤ 0.01), or ***(*p* ≤ 0.001) as determined by 2-way RM ANOVA with Sidak correction for multiple testing. Graphs were made by GraphPad Prism 9, version 9.0.0 (https://www.graphpad.com)

Finally, in order to evaluate possible additive and synergistic effects of antibiotics on the studied phenotypes, we performed a checkerboard assay with antibiotics at sub-inhibitory concentrations (concentrations summarized in Table 2). The experiment revealed that the cording phenotype was significantly more susceptible to the combination of rifampicin, linezolid or pretomanid in conjunction with other drugs as compared to the planktonic bacteria (Fig. 8).

**Table 1:** IC_50_ and MIC values for antibiotics (*n* = 3)

Antibiotic	IC_50_ [µg/ml, (CI)]		MIC (CI)/Standard MIC [µg/ml]^**^		Published MIC-ranges [µg/ml] [29, 30]
	Planktonic	Cording	Planktonic	Cording	H37Rv ATCC 27,294
Rifampicin	0.0013 (0.00083–0.0019)	0.00070 (0.00036–0.0013)	0.017 (0.0077–0.038)/0.016	0.0088 (0.0029-0.27)/0.016	0.06–0.25
Isoniazid	0.00074 (0.00031–0.0017)	0.00036 (6.9 × 10^− 5^-0.00082)	0.0021 (0.00069–0.0067)/0.004	0.0028 (0.0011–0.0069)/0.004	0.03–0.06
Linezolid	0.36 (0.12–1.1)	0.15 (0.065–0.36)	1.7 (*)/2	1.1 (*)/1	0.25-1
Levofloxacin	0.29 (0.15–0.55)	0.23 (0.12–0.44)	1.1 (*)/1	0.91 (*)/1	0.25-1
Ethambutol	0.97 (0.19–5.3)	0.77 (0.11–5.5)	8.0 (*)/8	6.4 (*)/8	1–4
Clofazimine	0.28 (0.034-*)	0.081 (0.0018-*)	0.31 (0.018–5.28)/0.5	ND	0.12–0.5
Moxifloxacin	0,019 (*-0.063)	0,0037 (*-0.032)	ND	ND	0.06–0.25
Pretomanid	0,073 (*)	0,037 (*-0.17)	ND	ND	ID

*ND* no value detected, *ID* insufficient data

*not enough data to detect one or both limits of confidential interval (95 %)

**MIC value transposed to nearest higher one according to the ISO standard

## Discussion

Our major goal in this study was to evaluate the possibility of using automated live-cell imaging system IncuCyte S3 for high-troughput screening of assessment of TB drug susceptibility and compound libraries. We included two distinct phenotypes of Mtb in our high-throughput screening assay and investigated the potential differences in their antibiotic susceptibility. We were able to detect differences between the phenotypes as change of frequency in sizes of mycobacterial aggregates and monitor their growth and response to antibiotics. We were also able to calculate IC_50_ and MIC values for RIF and INH and the other antibiotics tested.

Heterogeneity of mycobacterial phenotypes in infected tissues and changing microenvironment during the course of TB poses a challenge for successful treatment that eliminates all forms of the pathogen. As we and others have previously demonstrated, the cording phenotype has possible bearings on TB severity, as it contributes to the pathophysiology [8, 9] and causes MET formation in infected macrophages [6]. This prompted us to compare the effect of antibiotics on cording and planktonic mycobacteria.

The use of relevant models is crucial for transferring hits from *in vitro* assays to the actual treatment. Even if the main niche of Mtb is the intracellular environment, mycobacteria in general often prevail extracellularly as cords [27, 28] and such model completes the host-based systems. We reasoned that DMEM could reflect the host environment even if it may not be optimized for bacterial growth in vitro where enriched bacterial growth medium is used for routine microbiological testing. The presence of human serum in our cell culture medium rather than fetal calf or bovine serum further adds to the simulation of a relevant environment. Although the generation of planktonic and cording bacteria occurred in broth with or without Tween-80, the phenotypes were keeping their distinct character in the Tween-80-free cell culture medium we used afterwards.

Detergents like Tween-80 in mycobacterial cultures ensures the dispersion of cells and promotes a homogenous culture, since assays with ideally dispersed bacterial cells are more readily reproducible in drug-screening assays [31]. We found the variability of less than 20 % in our experimental model acceptable. The presence of detergent causes an artificial condition that can affect the growth characteristics and therefore also antibiotic susceptibility. It has been reported for bedaquiline, that antibiotic susceptibility, measured as MIC, changes with Tween-80 concentration [32], since the detergent can interact with the antibiotic or even penetrate mycobacterial cell walls as reviewed by Leisching et al. [33]. Phagocyte internalization of Mtb and the subsequent innate immune response was shown to be affected by the presence of detergent in the culture medium [31]. Assays with detergent-free Mtb cultures can thus provide better information of the potential *in vivo* efficacy of tested drugs.

The data enabled us to reliably determine IC_50_ and MIC values for RIF and INH. As MIC is an important reference standard in microbiology, the MIC determination allowed us to compare findings to previous studies Even though our MIC values were determined with slightly different definitions than in clinical practice, they were in the range of those reported for clinical isolates in BACTEC 960 MGIT [34, 35]. We found a slightly lower MIC of 0.016 mg/L for rifampicin which may be due to a lower inoculum in our experimental system compared to the reference method. No significant difference between the two phenotypes was found in MIC values when results for all antibiotics were combined. The difference in antibiotic susceptibility estimated by mathematical approximation disappeared also after the MIC values were transposed to their ISO standards. That indicates that it was a marginal difference and should be interpreted with caution. Although the absolute differences were modest, IC_50_ results showed significantly lower values for cording compared to planktonic phenotype. The cording phenotype also seemed to be more susceptible to pair-wise combinations of several antibiotics. As we could see both in our recent and previous [6] experiments, the total area of cording bacteria was increasing overtime at significantly greater rate than the area of the planktonic bacteria. It has been shown that cords consist of bacterial cells with smaller volume than cells in non-cording aggregates, which can indicate more active replication within the cord [9] and active replication has been often associated with higher antibiotic susceptibility [36]. More experimental evidence, including larger range of tested concentrations of antibiotics would be needed to confirm if there indeed is variation in antibiotic susceptibility between these two phenotypes.

The live-cell imaging system we used enabled automated collection of images in chosen intervals during whole experiment without disturbing the experimental model. Image-based analyses with their ability to visualize events are highly relevant for understanding of heterogeneity in bacterial populations and have thus potential to bring about a paradigm shift in our knowledge of bacterial behavior. High-throughput monitoring of single bacterial aggregates takes the analysis of heterogeneous systems to a new level, similarly to single-cells omics approaches.

## Conclusions

We validated the live-cell imaging system Incucyte S3 for high-throughput screening of two distinct phenotypes of Mtb. We were able to follow their growth dynamics and measure important parameters of drug activity such as IC_50_ and MIC values.

## Methods

### Bacterial culture

H37Rv (American Type Culture Collection, ATCC 27,294) harboring the pFPV2- plasmid encoding the green fluorescent protein (GFP) was grown and prepared as previously described [6]. In short, the bacteria were grown for 2–3 weeks at 37^o^C in Middlebrook 7H9 medium (BD Biosciences, USA) supplemented with 0.05 % Tween-80 and albumin-dextrose-catalase enrichment (ADC, Becton Dickinson) using 20 µg/ml of kanamycin (Sigma-Aldrich, MO) as a selective antibiotic. To generate two different phenotypes, the bacteria were reseeded as standing (planktonic) and shaken (cording) cultures before the experiment. The standing, plaktonic culture was passaged in a new medium with 0.05 % Tween-80 and incubated at 37^o^C for additional 5 days, while the shaken, cording culture was passaged in medium without Tween-80 and put on a shaker at 260 rpm 3 days prior to the experiment.

### Experimental protocol

The bacterial suspensions from both tubes (i.e. planktonic and cording) were prepared as described earlier [37]. Briefly, the bacterial suspensions were centrifuged twice at 5,000 x g for 5 min in phosphate-buffered saline (PBS) supplemented with 0.05 % Tween-80 and passaged through a sterile syringe equipped with 27-gauge needle to remove bacterial aggregates. After the final wash, bacterial pellets from both tubes were resuspended in antibiotic-free DMEM (Gibco) containing 25 mM HEPES (Gibco), 2 mM L-glutamine (Gibco) and 10 % active human serum (pooled from 5 healthy donors, blood bank of Linköping University Hospital) (ABF medium). The number of bacteria in each suspension was adjusted to same concentration (CFU/ml) as determined by measuring optical density (OD_600_) and described earlier [6]. Since ABF medium is commonly used in our lab in models of Mtb growing intracellularly in human cells and supports bacterial growth very effectively, we decided to continue cultivation in it after phenotypes have been generated in broth. Finally, 35 µl of each bacterial suspension was seeded in separate 384-well black clear-bottom plates (BD, Falcon) and placed in IncuCyte S3 (IncuCyte® Live-Cell Analysis System, Sartorius) for live cell imaging at 37^o^C for 48 h to allow initiation of growth in the DMEM. Images (2/well) at 20x magnification were captured with 2 h intervals. Selected first and second-line antibiotics available for TB treatment were dissolved either in sterile, deionized water or 100 % dimethyl sulfoxide (DMSO) to obtain stock solutions and then diluted in ABF medium to achieve the final required concentrations (summarized in Table 2). 35 µl of 2 times the final concentration of antibiotic solutions were added in respective wells after 48 h of addition of bacterial suspension to plate to make the final volume of 70 µl. The plate was then placed in IncuCyte S3 for additional 5 days (Fig. 1). The experiment layout was designed as such that one well was used for each antibiotic treatment and 33 wells were left for untreated controls on each plate and three replicated experiments were performed. Planktonic and cording bacteria were always seeded on separate plates. We also performed control experiment when Mtb was growing as standing culture at Middlebrook 7H9 broth supplemented with ADC for 2–3 weeks, passaged into fresh medium and after 6 days of growth filtered through 0,5 μm filter and seeded on 96-well plate in fresh medium with or without Tween-80.

**Table 2:** Solvent used in preparation of antibiotics and concentrations used in assays

Antibiotic	Solvent	Dose response ranges (µg/ml)	Checkerboard assay^a^ (µg/ml)
Rifampicin	DMSO	10–1 × 10^− 6^	1
Isoniazid	H_2_O	10–1 × 10^− 5^	0.1
Linezolid	DMSO	10–0.01	1
Levofloxacin	DMSO	10–0.01	1
Ethambutol	DMSO	40–0.04	4
Clofazimine	DMSO	10–0.001	10
Moxifloxacin	H_2_O	10–0.01	0.25
Pretomanid	DMSO	10–0.01	1

^a^subinhibitory concentrations

### Analysis of live-cell imaging data

Fluorescent objects representing planktonic and cording aggregates were identified with the help of inbuild IncuCyte S3 software enabling segmentation and background correction. Since GFP fluorescence, which in our bacteria is under the control of the Hsp60 promoter, is an unreliable parameter for detection, because many factors such as antibiotic treatment and other stressing factors could influence the expression levels, we exclusively used area of green fluorescent objects in our analyses. Data on area (µm^2^) of identified fluorescent objects was collected and exported from the IncuCyte S3 into Excel and GraphPad Prism 9 (version 9.0.0) for summarizing the results and statistical analysis. Differences in total area (per image) between untreated planktonic and cording controls over time were analysed by two-way RM ANOVA with Sidaks´s multiple comparison test assuming sphericity. Total area measurements after antibiotic treatment were normalized (Additional files 8–9) to the median of total area of untreated controls in each experiment and mean of three repetitive experiments with standard deviation in error bars were than used in further analysis. Difference between planktonic and cording bacteria exposed to combination of the antibiotics (checkerboard assay) were analysed by two-way RM ANOVA with Sidaks´s multiple comparison test. Intra- and inter-assay variability was calculated as previously published [38]. Variability of total area measurements within each plate (intra-assay variability) was calculated based on replicated (*n* = 33) samples of untreated Mtb on day 5. Variability of measurements between plates (inter-assay variability) was based on total area measurements of untreated Mtb at three separate experiments (first well of 33 replicated was chosen for each experiment) at the same incubation time. Both values were expressed as the coefficient of variation (CV%).

### Analysis of frequency of aggregate sizes

Data on frequencies of area measurements of fluorescent aggregates was extracted from IncuCyte S3 software. Size intervals were chosen so they were logarithmically distributed up to 10^4^µm^2^. Since some measurements had no objects present, those points were filtered out and median of replicated wells was used in further analysis. Differences in frequencies between untreated controls in planktonic and cording bacteria were analysed by multiple t-tests with correction for multiple comparisons using the Holm-Sidak method in GraphPad Prism 9 (version 9.0.0). Data from wells treated with antibiotics was after filtering analysed directly since there was only one well per treatment in each experiment. To compare frequency of aggregate sizes without and after treatment with antibiotics, a time course of the median frequency at each size category was plotted.

### Analysis of dose response to antibiotics and MIC values

As recommended by GraphPad Prism 9 (version 9.0.0), IC_50_ values were calculated by fitting data into dose reponse curves (inhibitor vs. response) by nonlinear regression with three parameters and standard slope for those antibiotics where only 4 or 5 concentrations were tested. Nonlinear regression with four parameters and variable slope was used in case of RIF and INH, where 13 concentrations were avalaible. MIC, defined as lowest concentration enough to effectively reduce the growth of bacteria relatively to control, was determined in GraphPad using modified Gompertz function [39]. The MIC was then transposed to nearest higher MIC value using the the log_2_-scale according to ISO-standard.

### Image acquisition, processing and statistical analysis of largest aggregates

To measure the growth of a single aggregate, images were analyzed through MATLAB (v R2017a) image processing with in-house scripts (Additional file 10, MATLAB code). Images were extracted from one single large aggregate identified in a well without antibiotics (Additional file 3: Movie S1). The area of the particular aggregate was measured over time and changes of the area were calculated using the formula,

$$∆\text{A}=\text{A}_{\text{i}+1}-\text{A}_{\text{i}};\text{and}\,∆\text{t}=\text{t}_{\text{i}+1}-\text{t}_{\text{i}}.$$

where ∆A is the change of area; ∆t is the change of time, and i is the unit vector.

## Supplementary Information

**Additional file 1: Figure S1.** Mtb aggregates in broth. Mtb was grown in Middlebrook 7H9 broth with or without Tween-80 as indicated for 6 days and images (20x) were taken using IncuCyte S3 live-cell imaging system. Scale bar represents 200μm. Images were made by Incucyte® Base Software, version 2019B Rev3 (https://www.essenbioscience.com).

**Additional file 2: Table S1.** Frequency of aggregate sizes in planktonic and cording bacteria. Data represents median values of 33 technical replicates of untreated controls for both planktonic and cording phenotype in three independent experiments. NP (non-present) marks intervals where no aggregates were identified. Number of events in each experiment >100.

**Additional file 4: Figure S2.** Growth of a single aggregate. The figure summarizes the growth of the single aggregate followed in Movie S1. Data is presented as area in pixels. Graph were made by MATLAB (v R2017a, https://se.mathworks.com).

**Additional file 5: Figure S3.** Comparison of aggregate area after exposure to rifampicin (RIF) and isoniazid (INH). Planktonic and cording phenotype were untreated or exposed to increasing concentration of RIF and INH and average area of fluorescent objects measured. Data is presented as mean of average of the aggregate area (μm^2^) ±SD (*n* = 3). Significant differences between planktonic and cording phenotype are indicated with **(*p*≤0.01), or ***(*p*≤0.001) as determined by 2-way RM ANOVA with Sidak correction for multiple testing. Graphs were made by GraphPad Prism 9, version 9.0.0 (https://www.graphpad.com).

**Additional file 6: Table S2.** Intra- and inter-assay variability. Intra- and inter-assay variability was calculated using replicated measurements (*n* = 33) of total area/image (μm^2^) from 3 replicated experiments. Data is presented as coefficient of variation CV (%).

**Additional file 7: Figure S4.** Growth rate of planktonic and cording bacteria. Growth of planktonic and cording bacteria was measured as a total area/image in μm^2^ (A) or as ratio of the total area over d0 (B). Data is presented as mean±SD (*n* = 3). Significant differences between planktonic and cording phenotype for both total area/image and ratio over day0 (A-B) were determined by 2-way RM ANOVA with Sidak correction for multiple testing and are presented as *p*-values in (C). Graphs were made by GraphPad Prism 9, version 9.0.0 (https://www.graphpad.com).

**Additional file 8: Figure S5.** Normalization of the growth measurements after exposure to rifampicin (RIF). The growth of planktonic and cording bacteria was measured as total area/image (μm^2^) and normalized to the median of measurements for all untreated controls (*n* = 33) within same experiment and same phenotype. Normalized data for highest (A-B) and lowest (C-D) concentration of RIF used in experiments is shown. Data is presented as ratios over untreated controls for each experiment. Graphs were made by GraphPad Prism 9, version 9.0.0 (https://www.graphpad.com).

**Additional file 9: Figure S6.** Normalization of the growth measurements after exposure to isoniazid (INH). The growth of planktonic and cording bacteria was measured as total area/image (μm^2^) and normalized to the median of measurements for all untreated controls (*n* = 33) within same experiment and same phenotype. Normalized data for highest (A-B) and lowest (C-D) concentration of INH used in experiments is shown. Data is presented as ratios over untreated controls for each experiment. Graphs were made by GraphPad Prism 9, version 9.0.0 (https://www.graphpad.com).

**Additional file 10.** MATLAB code.

## Data Availability

The datasets used and/or analysed during the current study are available from the corresponding author on reasonable request. The MATLAB code and the README files can also be obtained from (https://github.com/JD2112/ImageProcessingMATLAB).
