# Supplementary figures and images for: A high-throughput screening assay based on automated microscopy for monitoring antibiotic susceptibility of Mycobacterium tuberculosis phenotypes
Source: BMC Microbiol. 2021 Jun 5;21:167. doi: 10.1186/s12866-021-02212-3 (PMC8178828; doi:10.1186/s12866-021-02212-3)

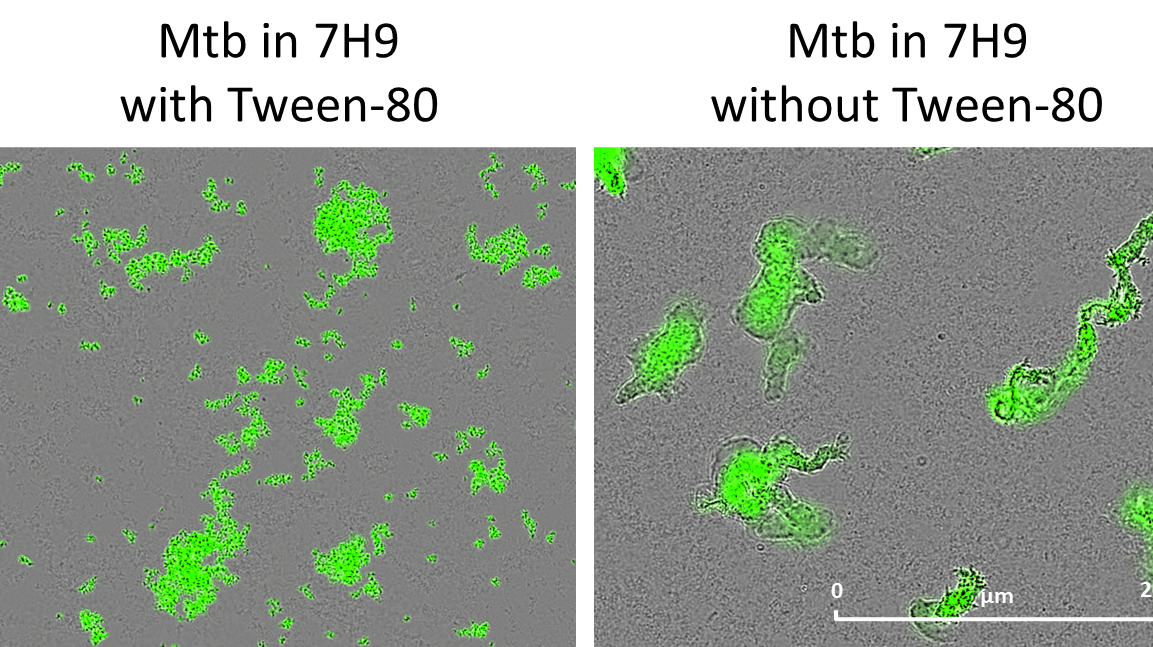

Supplement: Supplementary file 2 — Additional file 1: Figure S1. Mtb aggregates in broth. Mtb was grown in Middlebrook 7H9 broth with or without Tween-80 as indicated for 6 days and images (20x) were taken using IncuCyte S3 live-cell imaging system. Scale bar represents 200μm. Images were made by Incucyte® Base Software, version 2019B Rev3 (https://www.essenbioscience.com). [file 12866_2021_2212_MOESM1_ESM.tif]

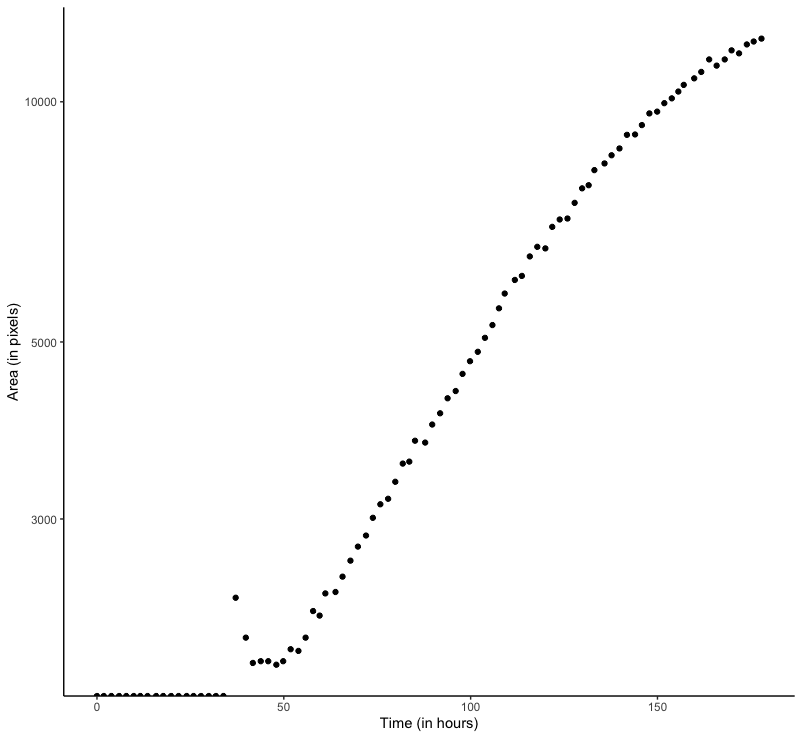

Supplement: Supplementary file 4 — Additional file 4: Figure S2. Growth of a single aggregate. The figure summarizes the growth of the single aggregate followed in Movie S1. Data is presented as area in pixels. Graph were made by MATLAB (v R2017a, https://se.mathworks.com). [file 12866_2021_2212_MOESM4_ESM.png]

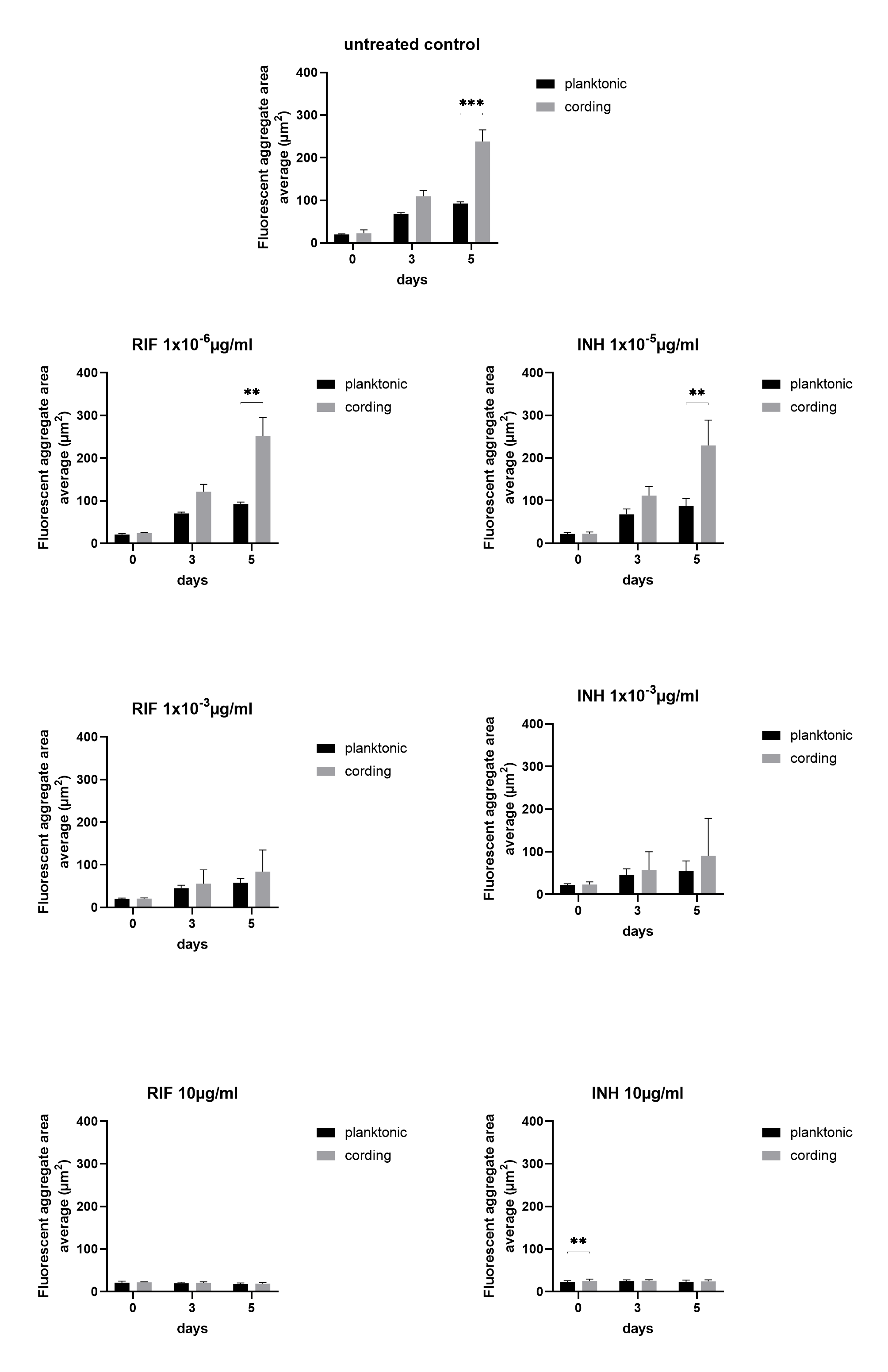

Supplement: Supplementary file 5 — Additional file 5: Figure S3. Comparison of aggregate area after exposure to rifampicin (RIF) and isoniazid (INH). Planktonic and cording phenotype were untreated or exposed to increasing concentration of RIF and INH and average area of fluorescent objects measured. Data is presented as mean of average of the aggregate area (μm2) ±SD (n = 3). Significant differences between planktonic and cording phenotype are indicated with **(p≤0.01), or ***(p≤0.001) as determined by 2-way RM ANOVA with Sidak correction for multiple testing. Graphs were made by GraphPad Prism 9, version 9.0.0 (https://www.graphpad.com). [file 12866_2021_2212_MOESM5_ESM.tif]

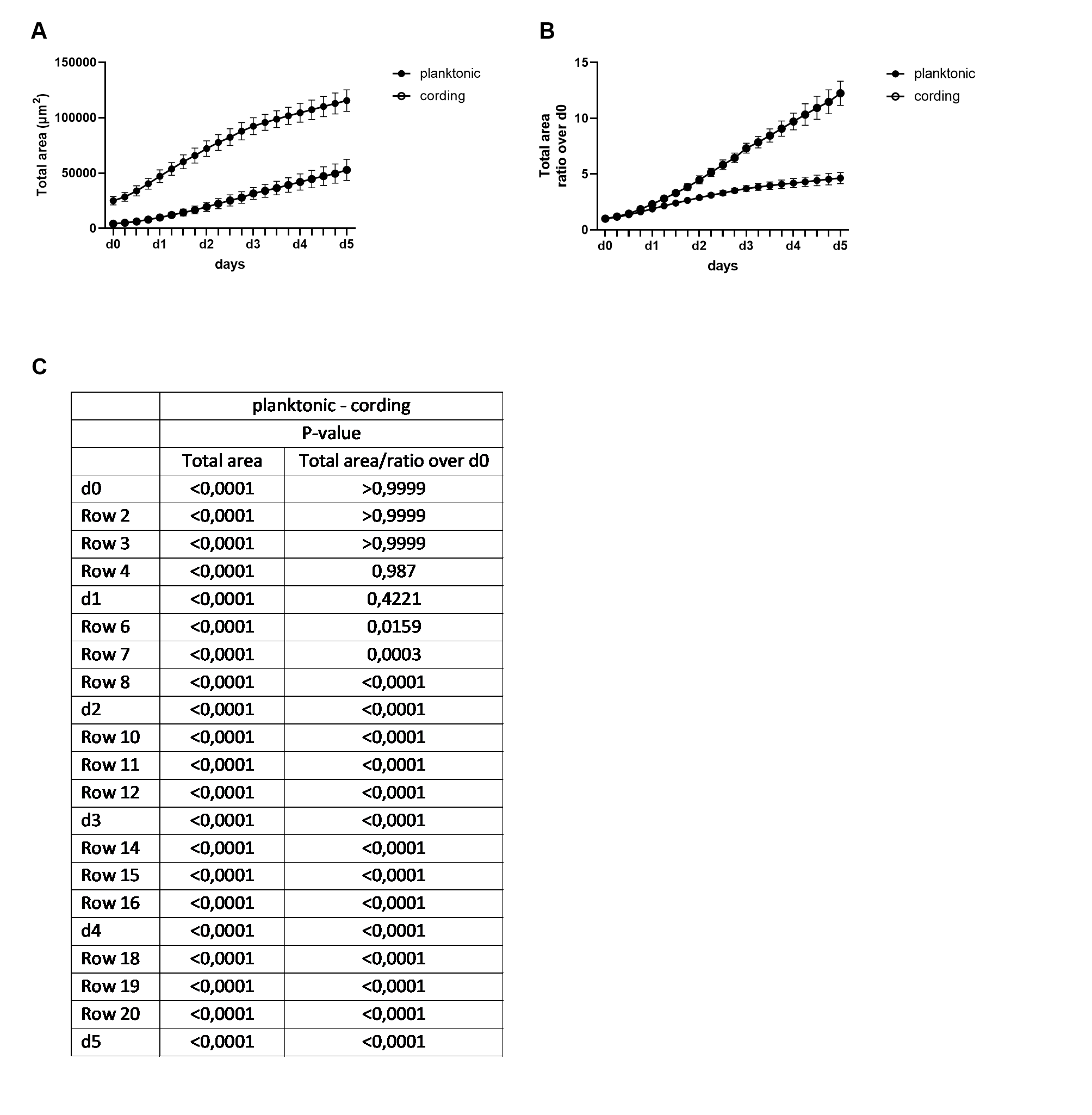

Supplement: Supplementary file 7 — Additional file 7: Figure S4. Growth rate of planktonic and cording bacteria. Growth of planktonic and cording bacteria was measured as a total area/image in μm2 (A) or as ratio of the total area over d0 (B). Data is presented as mean±SD (n = 3). Significant differences between planktonic and cording phenotype for both total area/image and ratio over day0 (A-B) were determined by 2-way RM ANOVA with Sidak correction for multiple testing and are presented as p-values in (C). Graphs were made by GraphPad Prism 9, version 9.0.0 (https://www.graphpad.com). [file 12866_2021_2212_MOESM7_ESM.tif]

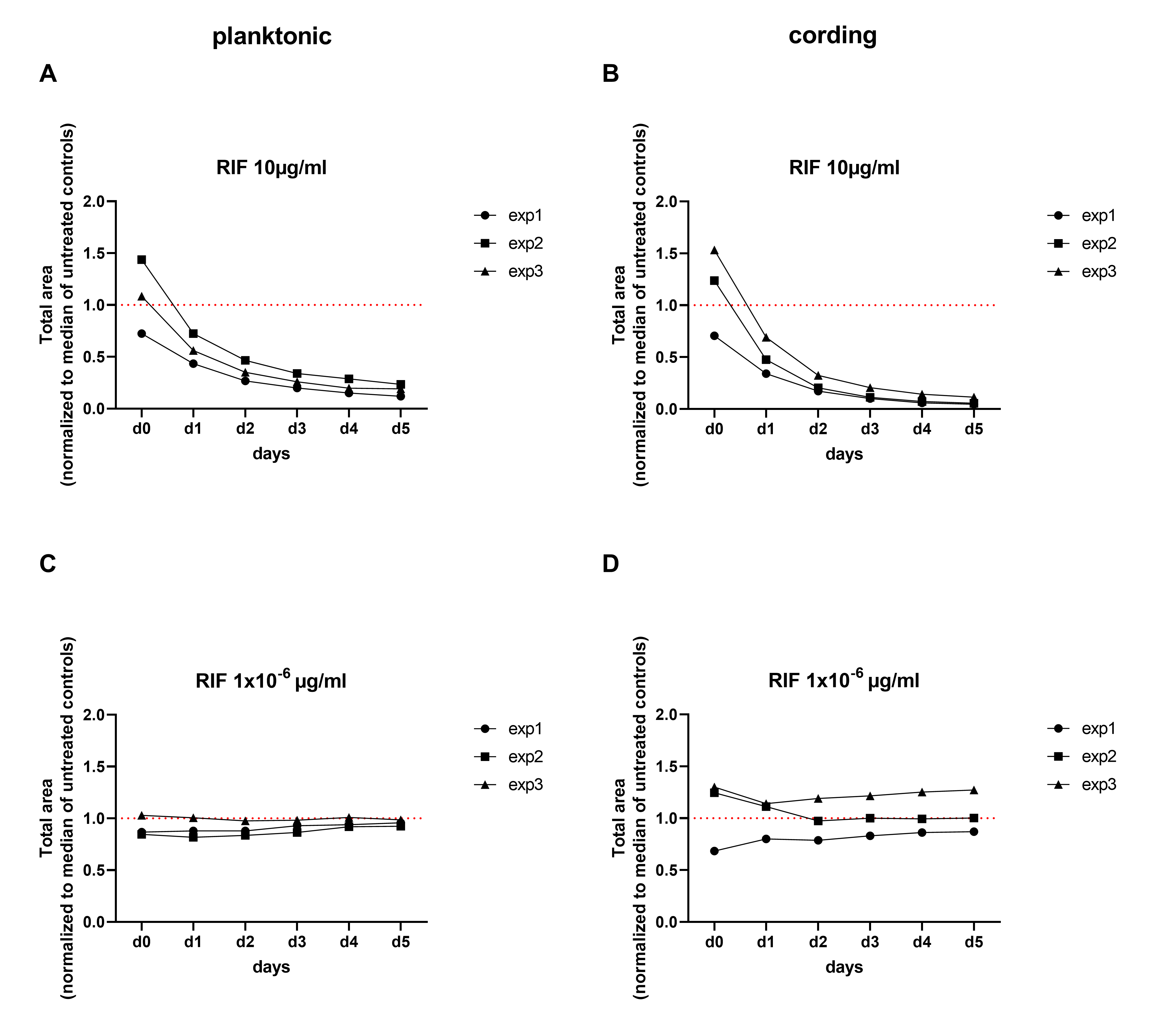

Supplement: Supplementary file 8 — Additional file 8: Figure S5. Normalization of the growth measurements after exposure to rifampicin (RIF). The growth of planktonic and cording bacteria was measured as total area/image (μm2) and normalized to the median of measurements for all untreated controls (n = 33) within same experiment and same phenotype. Normalized data for highest (A-B) and lowest (C-D) concentration of RIF used in experiments is shown. Data is presented as ratios over untreated controls for each experiment. Graphs were made by GraphPad Prism 9, version 9.0.0 (https://www.graphpad.com). [file 12866_2021_2212_MOESM8_ESM.tif]

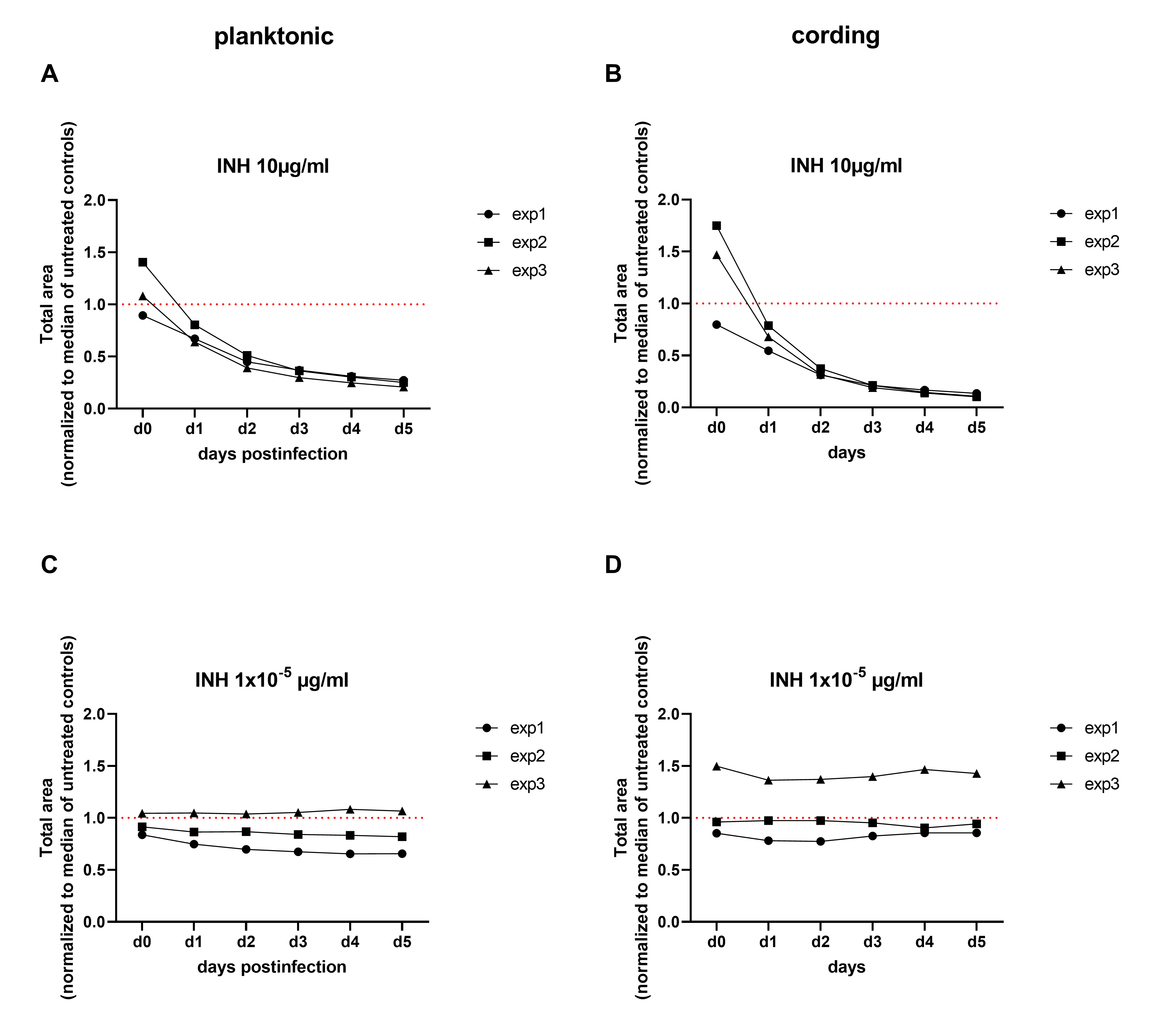

Supplement: Supplementary file 9 — Additional file 9: Figure S6. Normalization of the growth measurements after exposure to isoniazid (INH). The growth of planktonic and cording bacteria was measured as total area/image (μm2) and normalized to the median of measurements for all untreated controls (n = 33) within same experiment and same phenotype. Normalized data for highest (A-B) and lowest (C-D) concentration of INH used in experiments is shown. Data is presented as ratios over untreated controls for each experiment. Graphs were made by GraphPad Prism 9, version 9.0.0 (https://www.graphpad.com). [file 12866_2021_2212_MOESM9_ESM.tif]
