# Supplementary material for: A high-throughput screening assay based on automated microscopy for monitoring antibiotic susceptibility of Mycobacterium tuberculosis phenotypes
Source: BMC Microbiol. 2021 Jun 5;21:167. doi: 10.1186/s12866-021-02212-3 (PMC8178828; doi:10.1186/s12866-021-02212-3)
Supplement: Supplementary file 10 — Additional file 10. MATLAB code. [file 12866_2021_2212_MOESM10_ESM.pdf]

```
%%%%%%%%%%%%%%%%%%%%%%%%%%%%%%%%%%%%%%%%%%%%%%%%%%%%%%%%%%%%%%%%%%%%%%%%%%%%%%%%
%%%%%%%%%%%%%%%%%
%   This program will run in MATLAB and make a movie out of the image sequences
%   identified or stored in a Folder. The image sequences should be in a .tif
%   format file. The final image will be converted to .jpg files to make the
%   movie in .avi format.
%
%%%%%%%%%%%%%%%%%%%%%%%%%%%%%%%%%%%%%%%%%%%%%%%%%%%%%%%%%%%%%%%%%%%%%%%%%%%%%%%%%
%
% Test the figure files whether it has only one color channel or not
% Conditons of running the program is as follows
% 1. Please make sure you have all images in a particular folder
% 2. Images should be in .tif format, no other formats are supported by this
% codes.
% 3. The program is tested in Linux Mint 19.3, Mac OS 10.15, Windows 10 and MATLAB R2017a
% (acedemic version), MATLABR2019a. Other OS should be tested before the run.
%
%%%%%%%%%%%%%%%%%%%%%%%%%%%%%%%%%%%%%%%%%%%%%%%%%%%%%%%%%%%%%%%%%%%%%%%%%%%%%%%%
%%%%%%%%%%%%%%%%
clear all;
close all;
clc;

% Specify the folder where the files live.
myFolder = uigetdir('C:\'); %open the desired folder to run the code
%myFolder = '/home/jyoda68/Desktop/MATLABtest/';
% Check to make sure that folder actually exists.  Warn user if it doesn't.
    if ~isdir(myFolder)
            errorMessage = sprintf('Error: The following folder does not exist:\n%s', myFolder);
            uiwait(warndlg(errorMessage));
        return;
    end
% Get a list of all files in the folder with the desired file name pattern.

filePattern = fullfile(myFolder, '*.tif'); % Change to whatever pattern you need.

% read the files from the directory
theFiles = dir(filePattern);

% Making a new directory and transfer the modified images to that directory.
% Go to the directory of operation
cd(myFolder);

%mkdir ../MATLABtest mod_images;
% specify the directory where to save the files
  mod_images = fullfile(myFolder, 'mod_images');
% make a new directory to save the modified image files
mkdir(mod_images);

% Run the loop to copy all files for the modifications
for d = 1 : length(theFiles)
    baseFileName = theFiles(d).name;
        fullFileName = fullfile(myFolder, baseFileName);
            [~, filename,ext] = fileparts(fullFileName);
        newFileName = sprintf('%s_1%s', filename, ext);
    fprintf(1, 'Now reading %s\n', fullFileName);

% Now do whatever you want with this file name,
  % such as reading it in as an image array with imread()
  % Modify the images in the array and put the boundaries
    imageArray = imread(fullFileName);
        fd = im2double(imageArray);
            fdg = rgb2gray(fd);
                Iadj = imadjust(fdg);
                bw = imbinarize(Iadj);
            bw = bwareaopen(bw, 70);
        p = bwperim(bw);

    [B,L] = bwboundaries(bw);

    jd = figure;
    imshow(label2rgb(L, [0 1 0], [.1 .1 .1]));
        hold on

    for k = 1:length(B)
          boundary = B{k};
          plot(boundary(:,2), boundary(:,1), 'r', 'LineWidth', 2)
    end

  %imshow(imageArray);  % Display image.
  %drawnow; % Force display to update immediately.
  % save the images after the modifications in the new directory
saveas(jd, fullfile('mod_images', newFileName));
end

%%%-------------------------------------------------------------------------
% Making a new directory and transfer the modified images to that directory.
% myfolder = '/home/jyoda68/Desktop/MATLABtest/mod_images/';
% read the files from the modified folder
myfolder = fullfile(myFolder, 'mod_images');
  inputfilepattern = fullfile(myfolder, '*.tif');
tifthefiles = dir(inputfilepattern);

% Go to the folder
cd(myfolder);
  mkdir ../mod_images jpg;
% OutputFolder = fullfile('/home/jyoda68/Desktop/MATLABtest/mod_images/jpg/');
% create the output folder to save the modified files
OutputFolder = fullfile(myfolder, 'jpg');

% Run the loop to save all images
for n = 1:length(tifthefiles)
    baseFile = tifthefiles(n).name;
        fullFileNameInput = fullfile(myfolder, baseFile);
                rgbImage = imread(fullFileNameInput);
            fullFileNameOutput = fullfile(OutputFolder, baseFile);
        fullFileNameOutput = strrep(fullFileNameOutput, '.tif', '.jpg');
    imwrite(rgbImage, fullFileNameOutput);
end

%%%%---------------------------------------------------------------------
% ImageFolder = ('/home/jyoda68/Desktop/MATLABtest/mod_images/jpg/');
% Open image folder to crop the files in specific dimensions
ImageFolder = fullfile(myfolder, 'jpg');
  filePattern = fullfile(ImageFolder, '*.jpg');
jpegFiles = dir(filePattern);

% cd(ImageFolder);
for k = 1:length(jpegFiles)
  baseFileName = jpegFiles(k).name;
      fullFileName = fullfile(ImageFolder, baseFileName);
        fprintf(1, 'Now reading %s\n', fullFileName);
      imageArray = imread(fullFileName);
    imshow(imageArray);  % Display image.
  drawnow; % Force display to update immediately.
  % Get initial size
  [rows, columns, numberOfColorChannels] = size(imageArray);
      % Get size reduction / magnification factor
        %sizeFactor = 800 / columns;
          % Resize
            %newImage = imresize(imageArray, sizeFactor);
          % Get the new size.
        %[rows, columns, numberOfColorChannels] = size(imageArray);
      % Crop if necessary
  if rows > 200
    % Take upper 600 lines.  You could take lower or middle 600 also.
    newImage = imcrop(imageArray, [145 45 1400 1080]);
      newFileName = strrep(fullFileName, '.jpg', '_resized.jpg');
    imwrite(newImage, newFileName);
  end
end


%%%-----------------------------------------------------------------------
%ImageFolder = ('/home/jyoda68/Desktop/MATLABtest/mod_images/jpg/');
% Make the movie
filePattern = fullfile(ImageFolder, '*_resized.jpg');

  jpgFiles = dir(filePattern);

% Write the video in uncompressed file
  writerObj = VideoWriter('newMovie.avi', 'Uncompressed AVI');
% trying to set up the movie quality, default video quality 75
%writerObj.Quality = 80;
% write the desired frame rate, frames per second
  writerObj.FrameRate = 5;

% open the video file to write
open(writerObj);

% read the files and make the movie
for frameNumber = 1 : length(jpgFiles)
  baseFileName = jpgFiles(frameNumber).name;
    fullFileName = fullfile(ImageFolder, baseFileName);
      fprintf(1, 'Now reading %s\n', fullFileName);
    thisimage = imread(fullFileName);
  imshow(thisimage);
drawnow;
% write the video file
  writeVideo(writerObj, thisimage);
end

% close the video file to end the programs
close(writerObj);

%%%%%%%%%%%%%%%%%%%%%%%%%%%%%%%%%%%%%%%%%%%%%%%%%%%%%%%%%%%%%%%%%%%%%%%%%%%%%%%%%
% Conclude the program
% remove the temporary folders and files
% move the video file to the parent folder
  movefile *.avi ../;

% remove the temporary directory
rmdir jpg s;

% go to the parent directory
  cd ..;

% remove the other temporary directory
rmdir mod_images s;

% clear all the workspace and files
clear all;
  close all;
clc;

% exit from the program and from the MATLAB
%%% quit MATLAB

quit;
%%%%%%%%%%%%%%%%%%%%%%%%%%%%%%%%%%%%%%%%%%%%%%%%%%%%%%%%%%%%%%%%%%%%%%%%%%%%%%%%%
```
